# Engineering Multilayered Hepatic Cell Sheet Model Using Oxygen-Supplying MeHA/CPO Hydrogel

**DOI:** 10.3390/bioengineering12101132

**Published:** 2025-10-21

**Authors:** Kyungsook Kim, So Hee Han, Jiyoen Oh, Delger Bayarsaikhan, Moon Suk Kim, Dayoen Kim, Teruo Okano, Bonghee Lee

**Affiliations:** 1 Department of Biomedical Engineering, Jungwon University, 85 Munmu-ro, Goesan-eup, Goesan-gun 28023, Chuncheongbuk-do, Republic of Korea; 2 Cell Sheet Tissue Engineering Center (CSTEC), Department of Pharmaceutics and Pharmaceutical Chemistry, Health Sciences, University of Utah, 30 South 2000 East, Salt Lake City, UT 84112, USA; 3 Lee Gil Ya Cancer and Diabetes Institute, Gachon University, 155 Gaetbeol-ro, Yeonsu-gu, Incheon 21999, Republic of Korea; 4 Department of Molecular Science and Technology, Ajou University, Suwon 16499, Republic of Korea; 5 Institute of Advanced Biomedical Engineering and Science, Tokyo Women’s Medical University, 8-1 Kawada-cho, Shinjuku-ku, Tokyo 162-8666, Japan

**Keywords:** Cell sheet technology, 3D hepatic tissue, iPSC-derived hepatocytes, oxygen releasing hydrogel

## Abstract

Three-dimensional (3D) hepatic tissue engineering holds great potential for liver regeneration, disease modeling, and drug screening. These applications require densely layered hepatic tissues that mimic native 3D liver architecture. However, limited oxygen supply and reduced cell viability in densely layered hepatic constructs remain key challenges. To overcome this, this study developed a photo-crosslinkable, oxygen-releasing hydrogel composed of methacrylated hyaluronic acid (MeHA) and calcium peroxide (CPO). The MeHA/CPO hydrogel exhibited favorable rheological properties and sustained oxygen release. Induced pluripotent stem cell-derived hepatocyte (iHep) sheets were cultured with or without MeHA/CPO hydrogel in single- and double-layer formats. The hydrogel enhanced structural integrity and supported the formation of a multilayer (~33 µm). Double-layered iHep sheets with MeHA/CPO showed the significantly increased expression of paracrine factors (HGF, VEGF, Alb) and improved albumin secretion without loss of hepatocyte identity (AFP, HNF4α). This oxygen-releasing system effectively alleviates hypoxic stress, supporting the structural and functional viability of multilayered iHep sheets. Our platform provides a promising approach for engineering metabolically active hepatic tissues and may serve as a foundation for 3D hepatic tissue engineering.

## 1. Introduction

Liver diseases present a significant medical burden, ranking as the twelfth leading cause of death in the U.S. and fourth among middle-aged adults [1]. This burden continues to grow due to factors such as rising causes of non-alcoholic fatty liver disease (NAFLD) and steatohepatitis, the absence of a hepatitis vaccine, and an aging population vulnerable to hepatocellular carcinoma [2,3]. While orthotopic liver transplantation remains a definitive option for end-stage diseases, its limitations have motivated parallel efforts toward hepatocyte-based tissue engineering and an in vitro hepatic platform for disease modeling and pharmacological testing [4,5]. However, the therapeutic utility of autologous hepatocytes is fundamentally constrained by their limited availability and rapid phenotypic deterioration under conventional in vitro conditions. Recent advances in induced pluripotent stem cell (iPSC) technology have addressed several limitations by offering an ethically acceptable and virtually inexhaustible source of hepatocyte-like cells [6]. Although the potential risk of tumorigenesis in vivo remains, these cells exhibit hepatic functions and hold significant potential for disease modeling, pharmacological testing, and to inform regenerative applications [7,8,9]. Accordingly, to fully realize the potential of iPSC-derived hepatocytes (iHep) as in vitro 3D hepatic constructs and to inform future liver tissue engineering. It is critical to develop delivery platforms that preserve cell viability and functionality during fabrication.

Cell sheet engineering has garnered substantial attention as a scaffold-free strategy for fabricating 3D hepatic tissue constructs [10,11]. Utilizing temperature-responsive culture dishes, this technology enables the harvesting of contiguous cell sheets without enzymatic dissociation, thereby preserving extracellular matrix (ECM) components produced by the proliferating cells, cellular morphology, and intercellular junctions [10,12,13]. Notably, harvested cell sheets undergo 3D compaction and cytoskeletal remodeling, which enhance functional outputs relative to a flat monolayer [14]. Unlike traditional approaches that rely on exogenous biomaterials or adhesives, cell sheets possess intrinsic adhesion molecules that mediate spontaneous and stable integration between layers [14,15,16,17,18,19]. Our prior work successfully demonstrated the fabrication of stratified hepatic constructs by layering hepatocyte sheets with non-parenchymal cell sheets, such as endothelial cells, thereby enhancing hepatic polarity and functional maintenance [20,21]. However, direct stacking of multiple hepatocyte sheets failed to maintain cellular viability and functionality. Given the exceptionally high oxygen consumption rate of hepatocytes, we hypothesize that oxygen diffusion limitations within the multilayered constructs result in central hypoxia, leading to massive apoptosis and loss of function [22]. This observation underscores the need for innovative strategies to augment oxygen delivery in densely layered hepatocyte systems.

Various strategies have been explored to enhance oxygen availability, including the incorporation of oxygen-releasing compounds such as calcium peroxide (CPO) [23,24,25,26]. CPO has been widely investigated due to its ability to generate molecular oxygen through gradual decomposition; however, direct application often results in uncontrolled release and potential cytotoxicity [27,28]. Accordingly, we incorporated CPO into a biocompatible, photo-crosslinkable hydrogel matrix to achieve localized and sustained oxygen release. Methacrylated hyaluronic acid (MeHA), a chemically modified form of the naturally occurring glycosaminoglycan, was selected for its biocompatibility, biodegradability, and low immunogenicity [29]. These properties have supported its broad use in tissue engineering applications such as cartilage repair, wound healing, and drug delivery [29,30,31,32]. Functionalization with methacrylate groups permits photo-initiated crosslinking under mild ultraviolet (UV) irradiation, allowing precise spatiotemporal control over gel formation [29,31]. This property renders MeHA particularly suitable for in situ encapsulation or surface coating of cellular constructs because it enables rapid cross-linking without compromising cell viability and proliferation. In the present study, the formulation of the MeHA/CPO hydrogel was optimized to achieve mechanical robustness, rapid yet controllable gelation, and long-lasting oxygen generation. Subsequently, the effects of this oxygen-releasing platform on the viability, structural integrity, and hepatic function of iHep sheets were evaluated. By integrating this hydrogel with cell sheet engineering, we aimed to fabricate multilayered hepatic tissue constructs with enhanced cell survival and hepatic functionality, thereby overcoming a significant obstacle in three-dimensional hepatic tissue engineering.

## 2. Materials and Methods

### 2.1. Preparation of Calcium Peroxide-Crosslinked Meha Hydrogel

**Figure 1:**
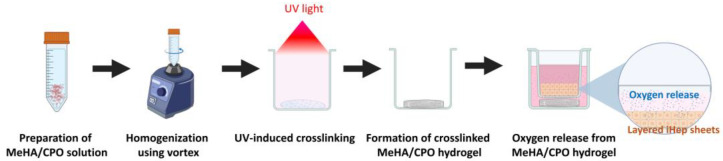
Schematic illustration of the preparation and application of MeHA/CPO hydrogel.

MeHA hydrogels were prepared at a final concentration of 1%, 3%, and 5% (*w*/*v*) by dissolving 15 mg, 45 mg, and 75 mg of MeHA (Sigma-Aldrich, St. Louis, MO, USA), respectively, in 1.5 mL of a 0.05% (*w*/*v*) 2-Hydroxy-4′-(2-hydroxyethoxy)-2-methylpropiophenone solution (Sigma-Aldrich) as a photo-initiator. The MeHA solutions were gently vortexed and transferred to glass containers. To incorporate CPO (Sigma-Aldrich) into each MeHA concentration, four different CPO concentrations (0.1%, 0.5%, 1%, and 5%) were combined to achieve 1.5 mg, 7.5 mg, 15 mg, and 75 mg of CPO per 1.5 mL MeHA solution. The twelve MeHA/CPO formulations were vortexed until homogeneously dispersed and subsequently crosslinked under ultraviolet (UV) irradiation (312 nm) for 5 min using a DAIHAN-brand Transilluminator UV (312 nm, WUV-L20, 230 V) (DAIHAN Scientific Co., Ltd., Wonju, Republic of Korea) (Figure 1).

### 2.2. Rheological Characterization of Crosslinked Meha Hydrogel

Initial tests showed that 1% MeHA failed to maintain stable gelation at CPO concentrations ≥ 0.5%, whereas 5% MeHA became too viscous for uniform CPO incorporation. In contrast, 3% MeHA maintained consistent gelation up to 0.5% CPO and was therefore selected as the representative formulation for rheological characterization. To evaluate the rheological properties, 3% MeHA and 3% MeHA containing 0.5% CPO were prepared as described above. The rheological measurements were performed using a rheometer (MCR 102) (Anton Paar, Ostfildern, Germany), with a 25 mm parallel plate geometry and a 0.5 mm gap maintained throughout the measurement. The temperature was set at 25 °C, and frequency sweeps were conducted over a 0.1–10 Hz range. For the crosslinked hydrogel groups, 400 µL of the precursor solutions were dispensed onto the inverted surface of a 60 mm Petri dish (Corning, New York, NY, USA), followed by UV irradiation. The 3% MeHA and the 3% MeHA containing 0.5% CPO groups were irradiated for 5 min. After crosslinking, the resulting gels were gently transferred to the rheometer platform using a flat spatula. For the non-crosslinked control groups, 400 µL of each precursor solution was directly loaded onto the rheometer plate without UV exposure, and measurements were performed immediately. We defined functional acceptance criteria as follows: rapid gelation within 5 min, (2) gel-like behavior within storage modulus (G’) consistently greater than loss modulus (G”) and tan δ < 1 across 0.1–10 Hz, and (3) a ≥ 500-fold increase in complex viscosity after UV exposure.

### 2.3. Oxygen Release Profile of Crosslinked Meha Hydrogel

To evaluate the oxygen-releasing capability of the crosslinked MeHA-based hydrogels, 200 mL of the hydrogel solution was added to each well and photo-crosslinked, forming a disk approximately 1 cm in diameter and 0.2 cm in thickness. 1× Phosphate-Buffered Saline (PBS) was carefully overlaid onto the hydrogel surface. The samples were then incubated under hypoxic conditions (5% O_2_) using a hypoxic incubator (SMA-30D) (ASTEC Co., Ltd., Fukuoka, Japan). After 0, 3, 6, 12, 24, 48, 72, 96, 120, 144, 168, 192, 216, 240 h of incubation, the dissolved oxygen concentration in the supernatant was quantitatively assessed using a pen-type dissolved oxygen meter (DO-9100) (Jinan Huiquan Electronic Co., Ltd., Jinan, China). For measurement, the sensor probe was vertically immersed in the 1X PBS overlaying the hydrogel, ensuring the electrode membrane was fully submerged without contacting the bottom of the well. Each sample was measured in triplicate to ensure analytical reproducibility.

### 2.4. Scanning Electron Microscopy (SEM) Analysis

To evaluate the incorporation and distribution of CPO particles within the MeHA hydrogel matrix, both MeHA and MeHA/CPO hydrogel samples (200 μL of hydrogel, forming disk-shaped specimens approximately 1 cm in diameter and 0.2 cm in thickness) were freeze-dried using a freeze dryer (Ilshin Bio Base Co., Ltd., Dongducheon, Republic of Korea). The freeze-dried specimens were mounted on aluminum stubs and sputter-coated with platinum. Surface morphology and CPO distribution were observed using a field emission scanning electron microscope (FE-SEM; SU8600, Hitachi, Tokyo, Japan) at an accelerating voltage of 5 kV.

### 2.5. In Vitro Cytotoxicity Assessment

iHeps, differentiated in this study from peripheral blood mononuclear cells (PBMC)-derived iPSCs obtained from WiCell (Madison, WI, USA), were employed to evaluate the cytocompatibility of oxygen-releasing MeHA-based hydrogels. The PBMC-derived iPSC working cell banks were prepared at passage 42. The iPSCs were seeded at a density of 4.5 × 10^4^ cells/cm^2^ in six-well tissue culture plates and differentiated into hepatocyte-like cells using a previously established protocol developed by our group [33,34]. Briefly, the cells were cultured in mTeSR™1 medium (STEMCELL Technologies, Vancouver, BC, Canada) supplemented with 5 μM ROCK inhibitor Y-27632 (STEMCELL Technologies) for 2 days. The stepwise differentiation included induction of definitive endoderm (DE) (3 days: DE stage) (5 days of differentiation), hepatic endoderm (HE) (5 days: HE stage) (10 days of differentiation), immature hepatocytes (IMH) (5 days: IMH stage) (15 days of differentiation), and mature hepatocytes (MH) (5 days: MH stage) (20 days of differentiation) using combinations of Activin A (R&D system), CHIR99021 (STEMCELL Technologies), bFGF (R&D System, Minneapolis, MN, USA), BMP4 (R&D System), HGF (Peprotech, Rocky Hill, NJ, USA), dexamethasone (Sigma-Aldrich), oncostatin M (R&D System), and DMSO (Sigma-Aldrich). At the end of the HE stage (day 10), cells were transferred to Transwell inserts (Falcon, Corning Inc., New York, NY, USA) at a density of 1.4 × 10^5^ cells per insert. After 1 day of culture in the MH stage (16 days), 200 μL of 3% MeHA hydrogel, with or without 0.5% CPO, was added to the bottom of 6-well plates. The hydrogels were photo-crosslinked under UV light for 5 min (Figure 1). The hydrogel volume was fixed at 200 mL after photo-crosslinking, forming a disk-shaped approximately 1 cm in diameter and 0.2 cm in thickness. The specimen dimensions were kept constant across all experiments to avoid variations in oxygen-release profiles.

Cytotoxicity was assessed at 1-, 3-, and 5-day MH stage (16, 18, 20 days of differentiation) using the Alamar Blue cell viability assay (Invitrogen, Carlsbad, CA, USA). All cell cultures were performed under normoxic conditions in an incubator. The reagent was diluted 1:10 in hepatocyte culture medium, and 1 mL of the working solution was added to the upper chamber of each insert after the insert was washed with PBS. Following a 1 h incubation at 37 °C, the supernatant was collected, and cells were rinsed and replenished with fresh medium. Absorbance was measured at 570 and 600 nm using a microplate reader (BioTek Epoch2) (Thermo Fisher Scientific, Waltham, MA, USA). All experimental conditions were analyzed in triplicate (*n* ≥ 3) to ensure statistical validity.

### 2.6. Fabrication of Single- and Double-Layered Cell Sheets

The dissociated endpoint of HE stage cells (10 days of differentiation) were seeded onto 35 mm temperature-responsive culture dishes (TRCDs) (SSCW G6; Cell Sheet Tissue Engineering Regenerative Medicine Initiatives (CSTERMi), Tokyo, Japan) coated with fetal bovine serum (FBS; Gibco, Waltham, MA, USA) overnight and maintained in stage-specific differentiation media until the mature hepatocyte (MH) stage for 7 days before detachment. To harvest cell sheets, the temperature was reduced to room temperature (20–25 °C), allowing spontaneous detachment of intact MH cell sheets within 30 min, without the need for enzymatic treatment and with minimal disruption to cell–cell and cell–matrix interactions. For experiments evaluating oxygen-releasing support, 200 mL of MeHA or MeHA/CPO hydrogel was photo-crosslinked on the bottom of the 6-well plates, forming a disk-shaped hydrogel approximately 1 cm in diameter and 0.2 cm in thickness, and the Transwell inserts were then placed above the crosslinked hydrogels. For mono-layered sheet preparation, a single detached MH cell sheet was carefully transferred to a 6-well Transwell insert pre-coated with FBS and incubated at 37 °C for 2 h to promote stable attachment. To construct double-layered cell sheets, a second MH sheet was placed on top of the first sheet after the initial incubation period. The adhesion between the two-layered cell sheets was confirmed by gentle pipetting, as the layers remained intact without separation. Owing to the abundant presence of cell adhesion proteins within the ECM of the sheets, spontaneous adhesion occurred without the need for external pressure or additional processing steps. The double-layered constructs were further incubated for 2 h at 37 °C to ensure complete integration before proceeding with downstream analysis after 5 days of culture. These cell sheet cultures were maintained under normoxic conditions.

### 2.7. Gene Expression Analysis

Gene expression analysis was performed on both mono- and double-layered cell sheets cultured under three conditions: control (no hydrogel), 0.5% CPO alone, and 3% MeHA/0.5% CPO hydrogel. These groups were selected to assess the impact of MeHA on the functional and phenotypic characteristics of the cell sheets compared with CPO alone. Total RNA was extracted from engineered cell sheets using TRIzol (Invitrogen) and the PureLink RNA Mini Kit (Life Technologies, Carlsbad, CA, USA) according to the manufacturer’s protocols. cDNA was prepared from 1 μg of total RNA using the High-Capacity cDNA Reverse Transcription Kit (Life Technologies, Carlsbad, CA, USA). qRT-PCR analysis was performed using Power SYBR Green PCR Master Mix (Life Technologies, Carlsbad, CA, USA) on an Applied Biosystems Step One instrument (Applied Biosystems, Waltham, MA, USA). Gene expression levels were assessed for the following genes, as listed in Table 1. Applied Biosystems manufactured all primers. Relative gene expression levels were quantified by the comparative CT method. Gene expression levels were normalized to those of the housekeeping gene GAPDH. Gene expression levels are relative to the monolayer control group. 

### 2.8. Albumin Secretion Assessment

To assess the effect of the oxygen-releasing hydrogel, 200 μL of 3% MeHA with or without 0.5% CPO was loaded into the bottom of a 6-well plate and crosslinked under UV light for 5 min. The inserts containing the single- and double-layered cell sheets were placed above the hydrogels and cultured in hepatocyte maturation medium with 3 mL in the lower chamber and 2 mL in the upper chamber. At 24 h post-media change, the supernatant was collected and centrifuged at 1000× *g* for 3 min to remove residual cell debris and floating particles before ELISA measurement. Albumin concentrations in the collected media were quantified using the Human Albumin ELISA Kit (Abcam, Cambridge, UK) following the manufacturer’s instructions. Absorbance was measured at 450 nm using a microplate reader (BioTek Epoch2) (Thermo Fisher Scientific).

### 2.9. Histological Analysis

For histological examination, the mono- and double-layered cell sheet constructs harvested as described in Section 2.6 were fixed with 4% paraformaldehyde for 30 min at room temperature. Following fixation, the samples were washed three times with 1× PBS, each for 5 min. For cryosections, samples were embedded in optimal cutting temperature (O.C.T.) compound (Leica Biosystems, Wetzlar, Germany). The cryosections were equilibrated to room temperature for 30 min, followed by post-fixation in 4% PFA for 15 min. After rinsing three times with PBS, slides were washed in tap water for 5 min. For hematoxylin and eosin (H&E) staining, slides were immersed in Mayer’s hematoxylin solution (Dako, Santa Clara, CA, USA) for 2 min and 30 s. The excess moisture was removed after rinsing in tap water for 3 min. The sections were then stained with eosin Y (Sigma-Aldrich) for 1 min and 30 s. Finally, slides were mounted using DPX mountant (Sigma-Aldrich). The quantitative analysis of cell sheet thickness was performed on H&E-stained cross-sections. For each group, three representative images were acquired. In each image, five random positions were selected to measure the vertical thickness of the cell sheet, resulting in a total of 15 measurements per group.

## 3. Results

### 3.1. Meha-COP Hydrogel Formation Behavior Varies Depending on Meha and CPO Concentrations

**Figure 2:**
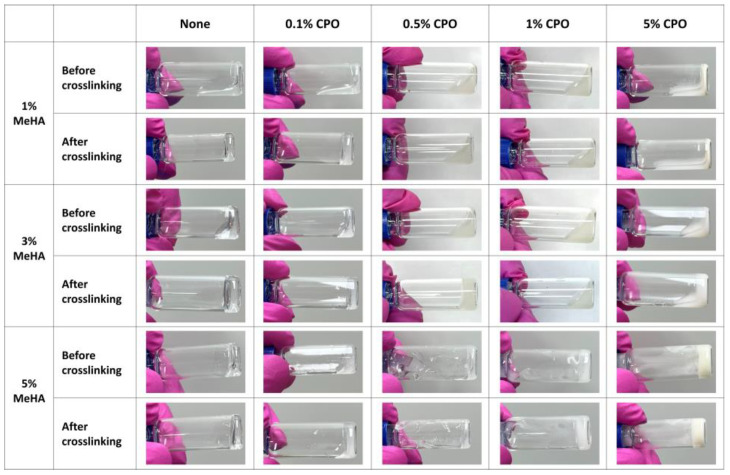
Macroscopic evaluation of MeHA hydrogel formation in the presence of varying CPO concentrations. Representative images showing the gelation behavior of MeHA with various concentrations (1%, 3%, 5%) and CPO concentrations (0%, 0.1%, 0.5%, 1%, 5%). Each formulation was observed before and after UV-induced crosslinking.

To investigate the gelation behavior of MeHA-based hydrogels, solutions of 1–5% MeHA were prepared in 0.05% photoinitiator. After vortexing for homogeneous dispersion, CPO was added at final concentrations ranging from 0.1% to 5%. UV crosslinking (312 nm, 5 min) was applied to induce hydrogel formation (Figure 2). Crosslinking was confirmed macroscopically by the formation of stable, self-supporting hydrogel after UV exposure, as shown in Figure 2, and no visible phase separation between the hydrogel matrix and CPO particles was observed even after immersion in PBS, indicating effective physical entrapment of the added CPO within the hydrogel network. In 1% MeHA hydrogels, effective crosslinking was observed only at a 0.1% CPO concentration, whereas gelation failed at concentrations of 0.5% or higher. In contrast, 3% of MeHA maintained stable gelation up to 0.5% CPO. For 5% MeHA, although crosslinking was visually confirmed at low CPO concentrations, the solution became too viscous to allow uniform incorporation of higher CPO levels. These results indicate that a 3% MeHA allows incorporation of higher CPO content while retaining gelation capacity, which was selected as the optimal formulation for subsequent experiments.

### 3.2. Crosslinking of Meha-CPO Hydrogel Induces Gel-like Rheological Behavior

**Figure 3:**
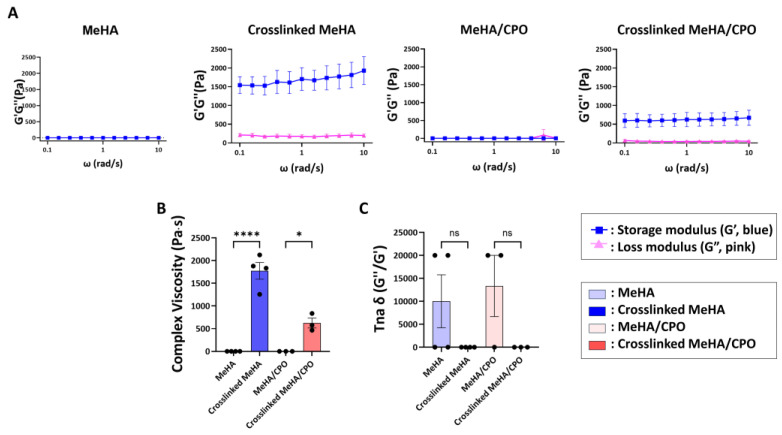
Rheological properties of MeHA and MeHA/CPO hydrogels before and after UV-induced crosslinking. (**A**) Frequency sweep analysis showing storage modulus (G’, blue) and loss modulus (G”, pink) for each group. MeHA and MeHA/CPO groups were not significantly different. Both crosslinked MeHA (*p* < 0.0001) and crosslinked MeHA/CPO (*p* < 0.001) showed significantly higher G’ than G”, as determined by multiple unpaired *t*-tests, indicating stable gel-like behavior. (**B**) Complex viscosity and (**C**) loss factor (Tan δ) of MeHA and MeHA/CPO hydrogels before and after crosslinking. Data are presented as mean ± SE (*n* ≥ 3). * *p* < 0.05; **** *p* < 0.0001; ns: not significant (*p* ≥ 0.05).

Rheological analysis was conducted to evaluate the viscoelastic properties of 3% MeHA hydrogels with or without 0.5% CPO, before and after UV-induced crosslinking. As shown in Figure 3A, both MeHA and MeHA/CPO formulations exhibited sol-like behavior before crosslinking, as indicated by similar values of the storage modulus (G’) and loss modulus (G”). Upon UV crosslinking, both groups showed a significant increase in G’, which exceeded G”, suggesting a transition to a gel-like state. Furthermore, the complex viscosity increased by more than 500-fold after crosslinking (Figure 3B), and the loss factor (tan δ) markedly decreased (Figure 3C), further confirming the formation of a stable hydrogel network. These results demonstrate that crosslinked MeHA/CPO hydrogels possess gel-like mechanical characteristics, supporting their potential application as sustained-release delivery matrices.

### 3.3. Oxygen-Releasing Performance of Meha/CPO Hydrogel

**Figure 4:**
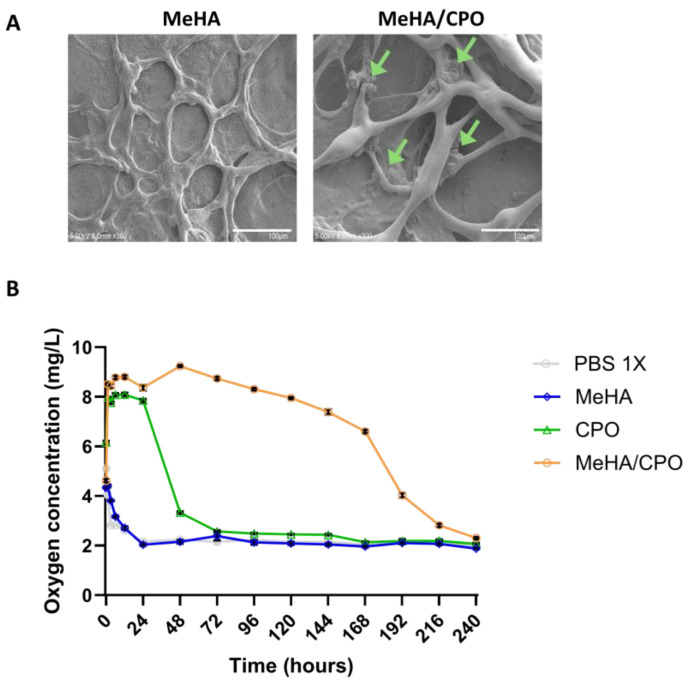
Characterization of MeHA/CPO hydrogel morphology and oxygen-releasing capability. (**A**) Scanning electron microscopy (SEM) images showing the porous microstructure of MeHA hydrogel and the presence of CPO particles (green arrows) embedded within the MeHA/CPO hydrogel. (**B**) Quantification of oxygen concentration in PBS, MeHA, CPO, and MeHA/CPO groups for 240 h (10 days). CPO and MehA/CPO groups showed a significant difference (*p* < 0.0001). Scale bars: 100 mm (**A**). Data are presented as mean ± SE (*n* ≥ 10) (**B**).

The hydrogel appeared visually uniform during gelation, suggesting an even dispersion of CPO in the MeHA matrix (Figure 2). SEM analysis of the cross-section of freeze-dried MeHA/CPO hydrogels revealed uniform distribution of CPO particles throughout the MeHA network, indicating successful encapsulation and homogeneous dispersion of the oxygen-releasing agent (Figure 4A).

To assess the oxygen-releasing performance, dissolved oxygen concentrations in the overlaid PBS were measured daily over 240 h (10 days) under hypoxic conditions (5% O_2_). As shown in Figure 4B, the MeHA/CPO group exhibited a higher dissolved oxygen concentration compared to MeHA alone, CPO alone, and PBS controls. Notably, dissolved oxygen levels in the MeHA/CPO group peaked at 8.8 mg/L at 24 h and remained elevated at 6.6 mg/L until 168 h (7 days). From 168 h to 240 h (10 days), oxygen levels gradually decreased. In contrast, the CPO group showed an initial increase in oxygen concentration during the first 24 h (1 day), followed by a rapid decline within 72 h (3 days). The MeHA and PBS groups consistently maintained levels at 2–3 mg/L throughout the observation period. These findings demonstrate that the photo-crosslinked MeHA/CPO hydrogel provides effective and prolonged oxygen delivery through stabilization of CPO within the hydrogel matrix.

### 3.4. The Meha/CPO Hydrogel Layer Did Not Adversely Affect the Morphology or Viability of Ipsc-Derived Hepatocytes

**Figure 5:**
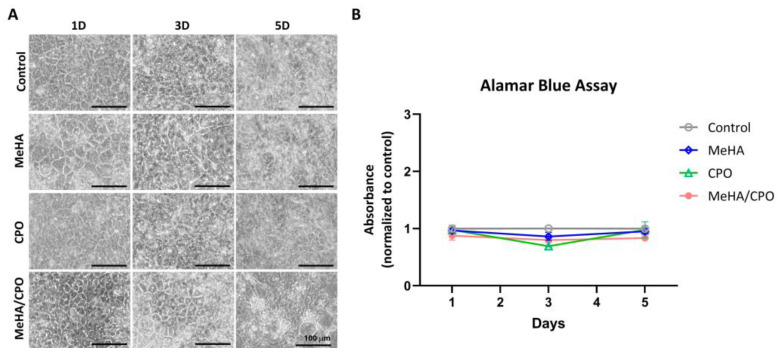
Effect of MeHA, CPO, and MeHA/CPO layers cocultured with iPSC-derived hepatocyte (iHep) morphology and viability. (**A**) Representative phase-contrast images of iHeps cultured with or without MeHA, CPO, or MeHA/CPO layers for 1, 3, and 5 days. (**B**) Cell viability was assessed using the Alamar Blue assay over 5 days. Scale bar = 100 µm (**A**). Data are shown as mean ± SE (*n* = 3) (**B**).

To assess the biocompatibility of MeHA, CPO, and MeHA/CPO formulations, hydrogel layers were formed on the bottom of 6-well plates, and iHeps were cultured on Transwell inserts placed above, allowing co-culture for 1, 3, and 5 days. As shown in Figure 5A, all treatment groups maintained a typical cuboidal morphology characteristic of hepatocytes throughout the culture period, indicating preservation of the hepatic phenotype. Cell viability was evaluated using the Alamar Blue assay (Figure 5B). No significant changes in metabolic activity were observed over the 5 days in any of the groups compared to the control. These results suggest that MeHA, CPO, and MeHA/CPO groups do not induce cytotoxic effects on iHeps and are biocompatible for use in hepatic cell culture systems.

### 3.5. Meha/CPO Hydrogel Enhances the Structural Integrity of Multilayered Ihep Sheets

**Figure 6:**
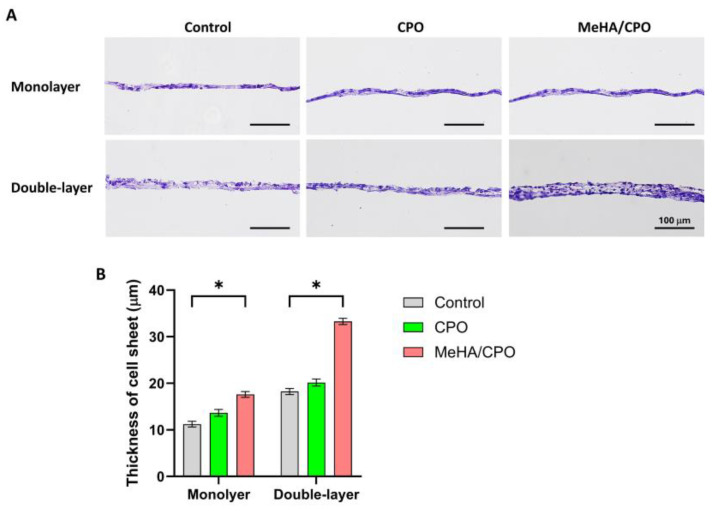
Effect of MeHA/CPO hydrogel on the structural integrity of monolayer and double-layer iPSC-derived hepatocyte (iHep) sheets. (**A**) Representative histological images of H&E-stained iHep monolayer and double-layer cell sheets after 5 days of culture with or without CPO or MeHA/CPO hydrogel layers. (**B**) Quantitative data on the thickness of layered cell sheets in control, CPO, and MeHA/CPO groups. Scale bar = 100 µm (**A**). Data are shown as mean ± SE (*n* = 3). * *p* < 0.05.

To evaluate the structural effect of MeHA/CPO hydrogel on monolayer and double-layer iHep sheets, multilayered constructs were generated by sequential stacking of cell sheets and cultured for 3 days with or without MeHA hydrogel. As shown in Figure 6A, H&E staining revealed that the double-layer sheets in the MeHA/CPO group maintained a more compact and organized structure, with clearly distinguishable nuclei and cytoplasmic boundaries compared to the control and CPO groups. Quantitative analysis of sheet thickness showed that double-layer sheets in the MeHA/CPO group had a significantly greater thickness (~33 µm) than controls and CPO groups, nearly doubling in size (Figure 6B). These groups (Control, CPO, and MeHA/CPO) were selected to determine whether the observed structural maintenance was due solely to the presence of CPO or to the sustained oxygen release provided by the MeHA-based hydrogel system. These findings indicate that MeHA/CPO hydrogel layer improves structural stability and integrity of multilayered hepatic cell sheets, likely by supporting cell–cell and cell–matrix interactions during culture.

### 3.6. MeHA/CPO Hydrogel Enhances Functional Activity of Multilayered iHep Sheets

**Figure 7:**
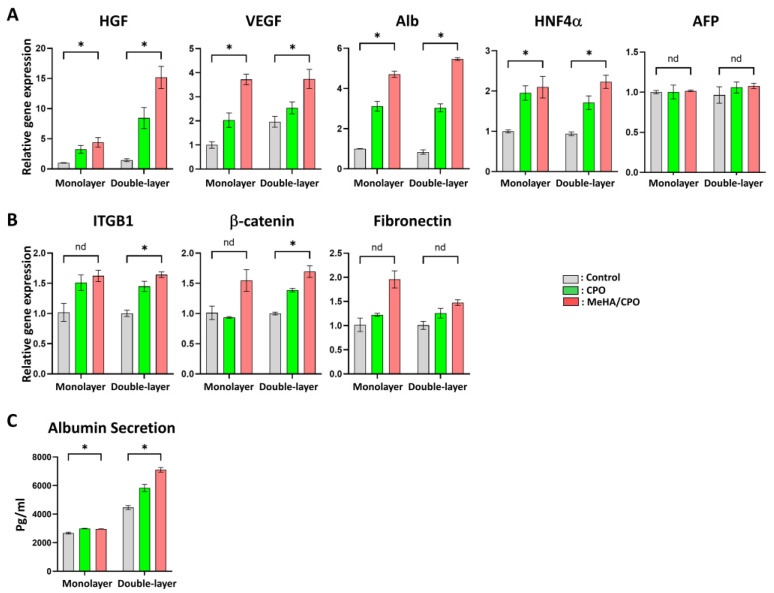
Effect of MeHA/CPO hydrogel on hepatic gene expression and albumin secretion in iHep sheets. (**A**,**B**) Relative gene expression levels of HGF, VEGF, Alb, HNF4α, AFP, ITGB1, β-catenin, and Fibronectin were analyzed by qPCR in monolayer and double-layer iHep sheets treated with Control, CPO, or MeHA/CPO hydrogels. (**C**) Albumin secretion was quantified by ELISA and presented as total secretion (pg/mL). Data are shown as mean ± SE (*n* = 3). * *p* < 0.05; nd, not detected.

To evaluate the functional characteristics of iHeps in different hydrogel conditions, gene expression and protein secretion analyses were conducted for three groups (Control, CPO, and MeHA/CPO). As shown in Figure 7A, quantitative PCR analysis revealed that HGF, VEGF, and Alb expression levels were significantly upregulated in the MeHA/CPO-treated double-layer iHep sheet group compared to the monolayer and other treatment groups. This enhancement is attributable to the oxygen-supplying effect of CPO within the hydrogel. In contrast, the expression levels of hepatic markers HNF4α and AFP remained unchanged across all groups, indicating the maintenance of hepatocyte identity regardless of treatment. As shown in Figure 7B, ITGB1 and b-catenin expression was elevated in the double-layer groups, with the highest levels in the MeHA/CPO-treated constructs. Fibronectin expression was not significantly different among groups but tended to be highest in the double-layer condition. These results suggest that the oxygen-releasing hydrogel supports intercellular junctions and extracellular matrix organization in the multilayered iHep sheets. Consistent with the gene expression data, ELISA results showed that albumin secretion, both in total amount, was significantly higher in the MeHA/CPO double-layer group compared to control group (Figure 7C). These results suggest that MeHA/CPO hydrogels support hepatocyte sheets’ structural and physiological integrity while enhancing their paracrine function.

## 4. Discussion

In this study, we developed an oxygen-releasing system based on MeHA hydrogels crosslinked by UV light for rapid gelation. The key technological advances of this study include: (1) the optimization of MeHA-CPO hydrogel formulation that ensures both gelation stability and oxygen-releasing capability, (2) the successful fabrication of layered iHep sheets supported by this hydrogel system, and (3) the enhanced secretion of bioactive molecules under sustained oxygen delivery without compromising hepatic identity. The following discussion elaborates on the significance of these findings in the context of hepatic tissue engineering and oxygen-sensitive cell-based therapies. In this study, the hydrogel was photo-crosslinked on the bottom of the culture wells, with iHep sheets maintained on transwell inserts above. MeHA did not provide mechanical support to the sheets but served only to stabilize CPO and enable sustained oxygen release. The difference between the CPO-alone and MeHA/CPO groups indicates that the improvement in sheet integrity and function derives from sustained oxygenation rather than from MeHA itself.

Numerous hepatic in vitro models have been developed for drug testing and disease modeling, including primary hepatocyte monolayers, spheroids, organoids, perfusion-based microfluidic chips, and scaffold-supported 3D constructs [1,35,36,37,38]. While these systems have advanced the field, they often face challenges such as loss of hepatic phenotype during long-term culture, limited paracrine activity, or hypoxia-related decline in function within dense constructs. Our approach, integrating iPSC-derived hepatocyte sheets with an oxygen-releasing MeHA/CPO hydrogel, provides a scaffold-free yet structurally stable platform [10,14] that maintains ECM and cell–cell junctions while alleviating hypoxia in multiplayer constructs, thereby complementing existing hepatic in vitro models. In the present study, we developed and optimized a CPO-incorporated MeHA hydrogel to be a biocompatible oxygen-releasing matrix for hepatic tissue engineering applications. The 3% MeHA formulation supplemented with 0.5% CPO demonstrated optimal crosslinking efficiency and oxygen-generating potential among the evaluated concentrations. This balance was critical, as excessive CPO loading hindered gelation. Although the addition of CPO partially reduced the viscosity of the MeHA hydrogel due to interference with crosslinking, the 3% MeHA/0.5% CPO formulation was chosen because it provided the most stable gelation while maintaining sufficient oxygen-releasing capacity. 0.5% CPO was used to assess hydrogel compatibility, and no cytotoxic effects were observed. However, at higher concentrations of CPO, potential ROS-related effects should also be considered. Rheological measurements further confirmed the successful photo-crosslinking behavior of this formulation, as evidenced by significant increases in storage modulus (G′) and complex viscosity following UV exposure, indicating a stable viscoelastic network appropriate for biomedical use. The rationale for engineering an oxygen-releasing platform originates from the high metabolic rate and oxygen demand intrinsic to hepatocytes. Quantitative assessments of oxygen consumption underscore this requirement. Balis et al. reported a peak oxygen uptake rate of 0.91 nmol O_2_/sec/10^6^ cells (~910 amol/sec/cell) in porcine hepatocytes during early culture stages [22]. In comparison, other cell types, including PC-3 prostate carcinoma cells (~44 amol/sec/cell) and WI-38 fibroblasts (~2.5 amol/sec/cell), exhibit significantly lower oxygen demands [39]. This disparity highlights the susceptibility of hepatocytes to hypoxic stress, particularly in multilayered or high-density constructs, where oxygen diffusion may be limited. Because direct numerical comparison is challenging, specifying a single numeric target was not feasible. Instead, the target was defined as the physiological range that preserves hepatocyte sheet viability and structural integrity. The MeHA/CPO hydrogel addresses this limitation by providing sustained, localized oxygenation through CPO decomposition, while maintaining a supportive, cell-friendly microenvironment. Although each originates as a monolayer, harvested cell sheets undergo 3D compaction while preserving ECM and intercellular junction, and when layered, they yield tissue-like assemblies consistent with prior observations [14]. Rapid in situ photo-crosslinking enables the hydrogel to bind to 3D hepatic sheets without compromising cell viability. These findings suggest that this platform has promise as an effective oxygen delivery system in advanced hepatocyte-based tissue constructs and may support broader applications in metabolically active tissue engineering models.

Hepatocyte function is critically influenced by cell–cell junctions and cell polarity, both readily lost under conventional monolayer culture conditions [40]. Previous studies have shown that hepatocytes cultured on temperature-responsive dishes can be harvested as continuous, intact sheets that retain cell–cell and cell–ECM interactions, making them suitable for constructing two- or three-dimensional hepatic tissue structures [41]. Cell sheet engineering has enabled the fabrication of stratified hepatic constructs, including co-culture systems with endothelial cell (EC) sheets to mimic in vivo hepatic microarchitecture and support hepatic function [20,21]. However, the stacking of hepatocyte sheets has historically been limited due to their high oxygen consumption rates and sensitivity to hypoxia. In the present study, we addressed this challenge by incorporating CPO into a photo-crosslinked MeHA hydrogel, providing sustained oxygen delivery directly to the hepatocyte sheet construct. This strategy enabled the successful stacking of two hepatocyte sheets without the need for auxiliary cell types. Notably, the double-layered hepatocyte construct maintained a thickness of ~33 µm with preserved cuboidal morphology, distinct intercellular junctions, and enhanced albumin secretion. These findings demonstrate that oxygen-releasing hydrogel systems can overcome the diffusion limitation of traditional multilayered hepatocyte cultures, supporting the formation of dense and functional 3D hepatic tissue constructs.

The functional capacity of hepatocyte constructs is determined by their viability and structural integrity, and their paracrine signaling activity, which plays an essential role in liver-specific function and regeneration [42,43]. In this study, double-layered iHep sheets cultured with MeHA/CPO hydrogel exhibited significantly elevated expression of key paracrine factors, including HGF, VEGF, and Alb, compared to the non-oxygen-supplemented group. These paracrine molecules are closely associated with liver repair and regeneration: HGF promotes hepatocyte proliferation and has anti-fibrotic effects, VEGF enhances angiogenesis and vascular stabilization within engineered tissues, and albumin synthesis reflects preserved hepatic metabolic function [44,45,46]. Therefore, the upregulation of these factors suggests that the constructs possess both regenerative and functional potential, supporting their applicability in treating liver injuries, promoting vascularization in grafts, and supplementing hepatic protein synthesis in cases of liver failure. These findings suggest that the sustained oxygen delivery from the CPO-containing hydrogel effectively alleviates local hypoxic stress in dense hepatic tissue constructs, thereby promoting enhanced secretory and regenerative functionality. In contrast, hepatic lineage markers, such as AFP expression, remained relatively stable across all conditions, with only minimal variation between the oxygen-supplemented and control groups. These results indicate that functional enhancement is achieved without compromising hepatocyte identity. Mechanistically, sustained oxygenation provided by the MeHA/CPO hydrogel appears to stabilize integrin-mediated cell-ECM adhesion and β-catenin-like junctions while preserving overall ECM organization, thereby supporting multiplayer integrity and function over time. Importantly, unlike prior approaches that relied on heterotypic co-culture systems to maintain hepatic phenotype under limited oxygen conditions, our strategy demonstrates that homotypic stacking of hepatocyte sheets is feasible when supported by engineered oxygenation. This preserves the hepatic phenotype and enhances paracrine activity and protein synthesis.

Although cross-sectional SEM suggested an even distribution of CPO, it provides only surface-level information, and further analyses will be needed to confirm the 3D distribution within the bulk hydrogel. Future studies incorporating computational modeling of oxygen diffusion and consumption within multilayered constructs would provide valuable quantitative insights and further strengthen our understanding of oxygen dynamics in 3D hepatic tissues. This proof-of-concept study demonstrates the feasibility of combining oxygen-releasing hydrogels with layered cell sheets to support hepatic cell viability. Further elaboration, including functional and long-term evaluations, is planned for future studies to advance this platform toward hepatic tissue engineering applications. In addition, optimization of formulation parameters such as the amounts of MeHA and CPO, photo-crosslinking conditions, and the incorporation of alternative oxygen-releasing systems (e.g., magnesium peroxide, perfluorocarbons, or enzyme-based carriers) or high-oxygen culture chambers to further benchmark and optimize oxygen delivery for constructing thicker and metabolically active 3D hepatic tissues. For the short-term (~5 days) thin-layer application used in the study, rheological measurements sufficed for integrity. However, to support longer-term (>14 days) applications, thicker and more highly crosslinked hydrogels together with complementary bulk testing will be required.

## 5. Conclusions

Integrating a photo-crosslinkable MeHA/CPO hydrogel with iPSC-derived hepatocyte sheet engineering enabled the fabrication of structurally stable and functionally enhanced multilayered hepatic constructs. This approach overcomes a significant barrier in hepatocyte-based tissue engineering by mitigating hypoxia-induced cellular stress through sustained oxygen delivery. These findings establish a versatile and biocompatible platform that can be broadly applied to develop dense, metabolically active tissue models for liver regeneration, disease modeling, and drug screening applications.

## Figures and Tables

**Table 1:** Primers used for gene expression analysis (qRT-PCR).

Target	Forward/Reverse (5′-3′)	Annealing Temp (°C)
HGF	F: 5′-GAG AGT TGG GTT CTT ACT GCA CG-3′	60.2
	R: 5′-CTC ATC TCC TCT TCC GTG GAC A-3′	60
VEGF	F: 5′-TTG CCT TGC TGC TCT ACC TCC A-3′	60
	R: 5′-GAT GGC AGT AGC TGC GCT GAT-3′	60
Alb	F: 5′-TGC CAA ACA GAG ACT CAA GT-3′	53.4
	R: 5′-TCA GCA GGC ATC TCA TCA TT-3′	53.4
HNF4a	F: 5′CAT GGC CAA GAT TGA CAA CCT-3′	56.2
	R: 5′-TTC CCA TATGTT CCT GCA TCA G-3′	56.4
AFP	F: 5′-ACA ATT CTT CTT TGG GCT GC-3′	53.4
	R: 5′-GCC ACA TCC AGG ACT AGT TT-3′	55.4
ITGB1	F: 5′-GGA TTC TCC AGA AGG TGG TTT CG-3′	60.2
	R: 5′-TGC CAC CAA GTT TCC CAT CTC C-3′	60
β-catenin	F: 5′-TGA GGA CAA GCC ACA AGA TTA C-3′	56.4
	R: 5′-TCC ACC AGA GTG AAA AGA ACG-3′	56.2
Fibronectin	F: 5′-ACA ACA CCG AGG TGA CTG AGA C-3′	60
	R: 5′-GGA CAC AACGAT GCT TCC TGA G-3′	60

## Data Availability

The data that support the findings of this study are available from the corresponding author upon reasonable request.

## Abbreviations

The following abbreviations are used in this manuscript:

3D	Three-dimensional
MeHA	Methacrylated hyaluronic acid
CPO	Calcium peroxide
iHep	iPSC-derived hepatocyte
PBS	Phosphate-buffered saline
HGF	Hepatocyte growth factor
VEGF	Vascular endothelial growth factor
Alb	Albumin
HNF4α	Hepatocyte nuclear factor 4 alpha
AFP	Alpha-fetoprotein
UV	Ultraviolet
FBS	Fetal bovine serum
EC	Endothelial cell
DE	Definitive endoderm
HE	Hepatic endoderm
IMH	Immature hepatocyte
MH	Maure hepatocyte
TRCD	Temperature-responsive culture dishes
